# A Prognostic Activity of Glutaredoxin 1 Protein (Grx1) in Colon Cancer

**DOI:** 10.3390/ijms25021007

**Published:** 2024-01-13

**Authors:** Marlena Brzozowa-Zasada, Adam Piecuch, Karolina Bajdak-Rusinek, Karolina Gołąbek, Marek Michalski, Natalia Matysiak, Zenon Czuba

**Affiliations:** 1 Department of Histology and Cell Pathology in Zabrze, Faculty of Medical Sciences in Zabrze, Medical University of Silesia in Katowice, 40-055 Katowice, Poland; 2 Department of Medical Genetics, Faculty of Medical Sciences in Katowice, Medical University of Silesia in Katowice, 40-055 Katowice, Poland; 3 Department of Medical and Molecular Biology, Faculty of Medical Sciences in Zabrze, Medical University of Silesia in Katowice, Jordana 19, 41-808 Zabrze, Poland; 4 Silesian Nanomicroscopy Centre in Zabrze, Silesia LabMed—Research and Implementation Centre, Medical University of Silesia, 40-055 Katowice, Poland; 5 Department of Microbiology and Immunology, Faculty of Medical Sciences in Zabrze, Medical University of Silesia in Katowice, Jordana 19, 41-808 Zabrze, Poland; zczuba@sum.edu.pl

**Keywords:** glutaredoxin 1, colorectal cancer, adenocarcinoma, ELISA, cancer cell lines, oxidative stress

## Abstract

Glutaredoxin 1 (Grx1) is an essential enzyme that regulates redox signal transduction and repairs protein oxidation by reversing S-glutathionylation, an oxidative modification of protein cysteine residues. Grx1 removes glutathione from proteins to restore their reduced state (protein-SH) and regulate protein-SSG levels in redox signaling networks. Thus, it can exert an influence on the development of cancer. To further investigate this problem, we performed an analysis of Grx1 expression in colon adenocarcinoma samples from the Polish population of patients with primary colon adenocarcinoma (stages I and II of colon cancer) and those with regional lymph node metastasis (stage III of colon cancer). Our study revealed a significant correlation between the expression of Grx1 protein through immunohistochemical analysis and various clinical characteristics of patients, such as histological grade, depth of invasion, angioinvasion, staging, regional lymph node invasion, and PCNA expression. It was found that almost 88% of patients with stage I had high levels of Grx1 expression, while only 1% of patients with stage III exhibited high levels of Grx1 protein expression. Furthermore, the study discovered that high levels of Grx1 expression were present in samples of colon mucosa without any pathological changes. These results were supported by in vitro analysis conducted on colorectal cancer cell lines that corresponded to stages I, II, and III of colorectal cancer, using qRT-PCR and Western blot.

## 1. Introduction

Colorectal cancer (CRC) is the third most frequent cancer in the world [1]. Diagnosing cancer at an early stage is essential, as the five-year relative survival rate for patients with stage I colorectal cancer is approximately 92%, compared to less than 10% for those with stage IV disease [1]. The survival of colorectal cancer patients has not improved significantly despite the availability of screening tests such as fecal occult blood testing and colonoscopy [2]. Unfortunately, the incidence of CRC is increasing in young adults due to several factors, such as a westernized diet, chronic stress, and lack of physical activity. Studies have indicated that 10% to 20% of all CRC patients have a positive family history, whereas approximately 5% of all CRC cases are associated with a known hereditary CRC syndrome that can be detected through germline testing [3,4].

According to the high capability to rapidly reproduce and adapt to various external conditions and cytotoxic treatments, cancer cells have a high metabolic demand. Reactive oxygen species (ROS) play a significant role in this process. ROS are generated when electrons released from fuel oxidation disrupt the balance of electron acceptor pairs, such as NADH/NAD+ and NADPH/NADP+. Electrons that are lost during fuel oxidation can combine with oxygen to form ROS [5]. Cells have developed various mechanisms to safeguard themselves from ROS, which can cause harm to their DNA, proteins, and lipids. One of the key pathways involved in counteracting ROS is glutathione (GSH). GSH is present in every cell compartment at concentrations ranging from 1 to 10 mM, and it plays a critical role in replenishing both enzymatic and non-enzymatic antioxidants. Glutathione peroxidases (GPX) participate in the oxidation of lipid hydroperoxides and H_2_O_2_, while glutathione S-transferase (GST) enzymes utilize GSH to detoxify exogenous substances or products induced by oxidative stress [6,7,8]. Other important enzymes associated with GSH metabolism are γ-Glutamyl transpeptidases (GGT). They are located on the outside of the cell surface and catalyze the hydrolysis of extracellular GSH. This provides the cell with a source of cysteine, which leads to an increased synthesis of intracellular GSH [9,10,11]. The enhanced levels of GGT have been found in cancers of the ovaries, colon, liver, melanoma, and leukemias [12,13,14,15,16]. In studies on melanoma cells in vitro and in vivo, elevated GGT activity has been found to accompany increased invasive growth, and a positive correlation has been described between GGT expression and unfavorable prognostic signs in human breast cancer [17,18,19]. The resistance of cancer cells expressing GGT could be due to the modulatory effects of GGT-mediated pro-oxidant reactions. These reactions regulate the signal transduction pathways that control the balance between proliferation and apoptosis. Additionally, they induce protective adaptations in the pool of intracellular antioxidants [20].

Cancer cells can adapt themselves to changes in redox balance also by the mechanism known as S-glutathionylation. This is a process where glutathione’s cysteine binds to the -SH (thiol) group of a target cysteine in the protein through a disulfide bond, and it oxidizes the target cysteine [21,22]. This modification is reversible and can lead to temporary changes in the function and structure of the target protein. Kinetic considerations suggest that glutaredoxins (Grx), a group of enzymes involved in the glutathionylation cycle, are crucial. The primary function of glutaredoxins is to deglutathionylate proteins that are already glutathionylated at a basal GSH/GSSG ratio [23]. S-glutathionylation has been shown to alter proteins involved in the signaling mechanisms of cancer cells. Protein kinase C (PKC) isozymes, which link multiple cellular processes to cancer, are particularly susceptible to oxidants due to their cysteine-rich regions [24,25]. Many transcription factors have conserved cysteine residues that are prone to thiol modifications. The S-glutathionylation of these transcription factors may disrupt their ability to bind to DNA, leading to the dysregulation of gene expression. The S-glutathionylation of cysteines within its proximal DNA-binding domain inhibits p53, a tumor suppressor known for its role in cell cycle control and apoptosis [26,27].

Among glutaredoxins, Glutaredoxin 1 (Grx1) is a major enzyme regulating redox signal transduction that repairs protein oxidation by reversing the oxidative modification of protein cysteine residues [28,29,30]. Grx1 has been shown to regulate the transcription nuclear factor-1 (NF-1) and RAS GTPase/MAP kinase, the contractile protein actin, and transcription factor cJun by restoring the original functional state of these modified proteins [31,32,33,34,35].

Unfortunately, there is little data in the literature on the role of Grx1 in carcinogenesis, particularly in the gastrointestinal tract, which is known to be highly exposed to ROS, oxidative stress, and thiol stress. Therefore, our study aimed to determine the immunohistochemical expression of Grx1 protein in samples of colon adenocarcinoma. We focused on patients with stages I, II, and III disease. It should be pointed out that most patients with colorectal cancer are diagnosed at an advanced stage, and the search for markers for early diagnosis and patient treatment is needed. Therefore, the choice of our study group seems justified. The results of the immunohistochemical analysis were correlated with the clinical data of the patients and their survival time. In addition, we wanted to verify the localization of the Grx1 protein in tumor tissue, which probably could be the basis for the studies associated with the development of targeted cell therapy in the future. Given that cancer tissue is a very heterogeneous environment, we also decided to carry out in vitro studies using cell lines to confirm the expression of *GRX1* gene strictly only in cancer cells without any exposition to signals from other parts of the tumor microenvironment. We utilized qRT-PCR and Western blot techniques for this purpose. The results of these studies will serve as a basis for further studies in which we intend to perform molecular studies related to *GRX1* gene expression. At this stage of our studies, we would like to verify whether the results obtained using immunohistochemical techniques in patients and samples of colon adenocarcinomas correlate with those obtained in vitro in the corresponding cell lines from stages I, II, and III patients. Importantly, our study was complemented by the ELISA analysis of Grx1 serum levels in patients. These data were also correlated with patient survival and clinical data.

## 2. Results

### 2.1. A Description of the Studied Group

The study involved 135 participants, comprising 65 men and 70 women. The average age of the participants was 64 years, with an age range of 55 to 77 years. Among all the cases, 63 (46.67%) had colon cancer on the right side, while 72 (53.33%) had it on the left side. The histological grades of differentiation were categorized into three types: G1 with 21 cases (15.55%), G2 with 72 cases (53.33%), and G3 with 42 cases (31.11%) (Table 1). 

**Figure 1:**
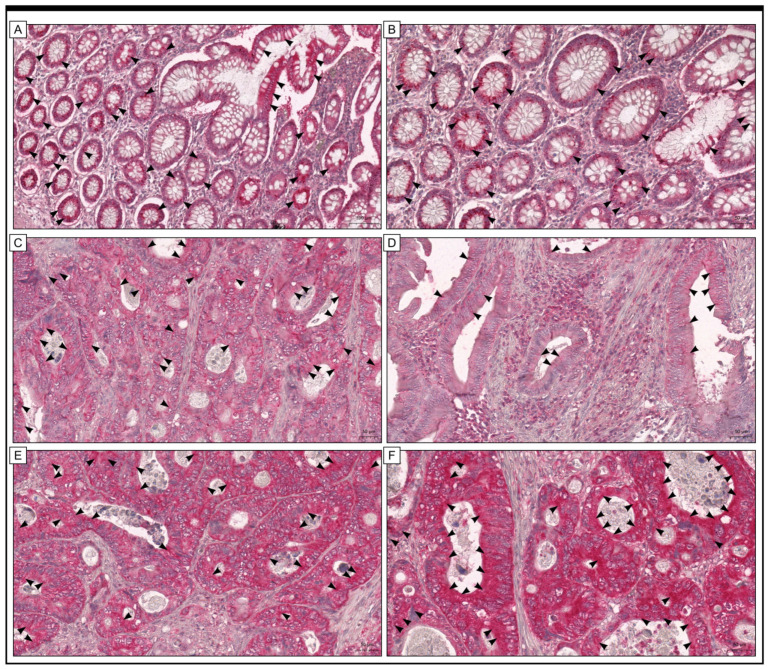
The expression of Grx1 was examined in samples of colon adenocarcinoma (**C**–**F**) and adjacent non-cancerous tissue margins (**A**,**B**). The black arrows indicate the expression of Grx1 in cells within the colon adenocarcinoma tissue, as well as within the cells of non-pathological mucosa. The scale bar shown in images (**A**–**F**) corresponds to 50 µm.

The Grx1 protein was found in both cancer and stromal cells in colon adenocarcinoma samples. The positive, high expression was also detected in colon mucosa without any pathological changes. Of the group studied, 34 samples of colorectal adenocarcinoma (25.19%) had high levels of Grx1 protein expression, while 101 samples (74.81%) had low levels of Grx1 protein expression (Figure 1).

### 2.2. Clinically Relevant Parameters (as Independent Variables) Correlated with Grx1 Immunohistochemical Expression

Following immunohistochemical analysis, the results were compared with the clinicopathological features of the patients. Moreover, we also showed the changes in the patient’s survival during five years. The study showed that Grx1 expression was significantly correlated with the histological grade of the tumor (*p* < 0.001, Chi^2^ test). The Grx1 was highly expressed in 13 samples (61.90%), 16 samples (22.22%), and 5 samples (11.90%) of G1, G2, and G3 tumors, respectively. In contrast, the low immunohistochemical expression of Grx1 protein was found in 8 samples (38.10%), 56 (77.78%), and 37 (88.1%). It is worth noting that there was a significant correlation between Grx1 expression and angioinvasion. In patients with positive angioinvasion, only 19.81% had high Grx1 immunohistochemical expression, while 80.19% had low immunoreactivity. Meanwhile, in patients without angioinvasion, 44.83% had high Grx1 expression, while 55.17% had low Grx1 immunoreactivity. Grx1 protein expression was also related to the depth of invasion (T). In T1/T2 patients, 62.86% had a high level of immunohistochemical reaction, while 37.14% had a low level of expression. In T2/T3 patients, only 12% had a strong immunohistochemical reaction for Grx1, while 88% had a low level of expression. The expression of Grx1 was also correlated with the expression of PCNA (*p* < 0.001, Chi^2^ test) and regional lymph node involvement (*p* < 0.001, Chi^2^ test). 

According to the study, the majority of patients with high PCNA expression had low Grx1 expression (86.02%), while only a small percentage had high Grx1 expression (13.98%). In the N1/N2 group, almost all patients had low Grx1 expression (96.15%), whereas high expression was uncommon (3.85%). In contrast, patients without lymph node invasion (N0) showed a relatively even distribution, with 57.39% exhibiting high Grx1 immunoexpression and 45.61% with low expression. Interestingly, 87.50% of stage I patients had high Grx1 expression, while the majority of stage II patients had low expression (66.67%). In stage III patients, high immunoreactivity was rare (1.33%), with low expression being the norm (98.67%) (*p* < 0.001, Chi^2^ test) (Table 2).

A statistically significant relationship was found when PCNA and the immunohistochemical expression of Grx1protein were treated as dependent variables. In the study group, there were more cases with both high levels of PCNA and low levels of Grx1 proteins (59.26%) of the total than there were subjects with both low levels of PCNA and high levels of Grx1 (15.56%). At the same time, the study group had a comparable number of people with low PCNA expression and low Grx1 protein levels (15.56%) and patients with high immunohistochemical expression of PCNA and Grx1 (9.635) (Table 3).

### 2.3. Prognostic Role of Grx1protein Expression in Colon Adenocarcinoma

**Figure 2:**
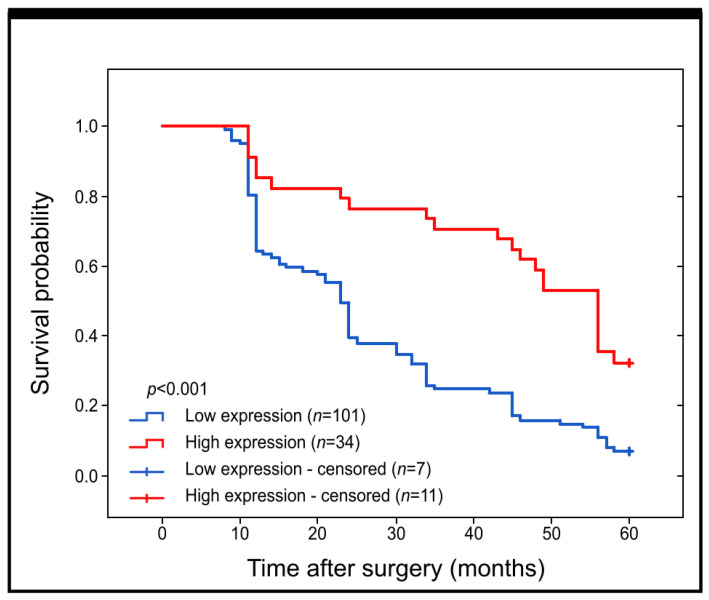
Kaplan–Meier curves showing the survival probability and its changes within the 60 months of the follow-up period for patients with high versus low expression of Grx1 protein in samples of colon adenocarcinoma tissues.

Our research aimed to investigate whether the presence of Grx1 protein has any effect on the survival rate of patients with colon adenocarcinoma, with a specific focus on its association with 5-year survival. We used Kaplan–Meier survival curves to analyze all samples. Our findings indicate that patients with higher expression of Grx1 had a significantly better 5-year survival rate than those with lower levels of this protein detected through immunohistochemical reaction (log-rank, *p* < 0.001) (as depicted in Figure 2).

**Figure 3:**
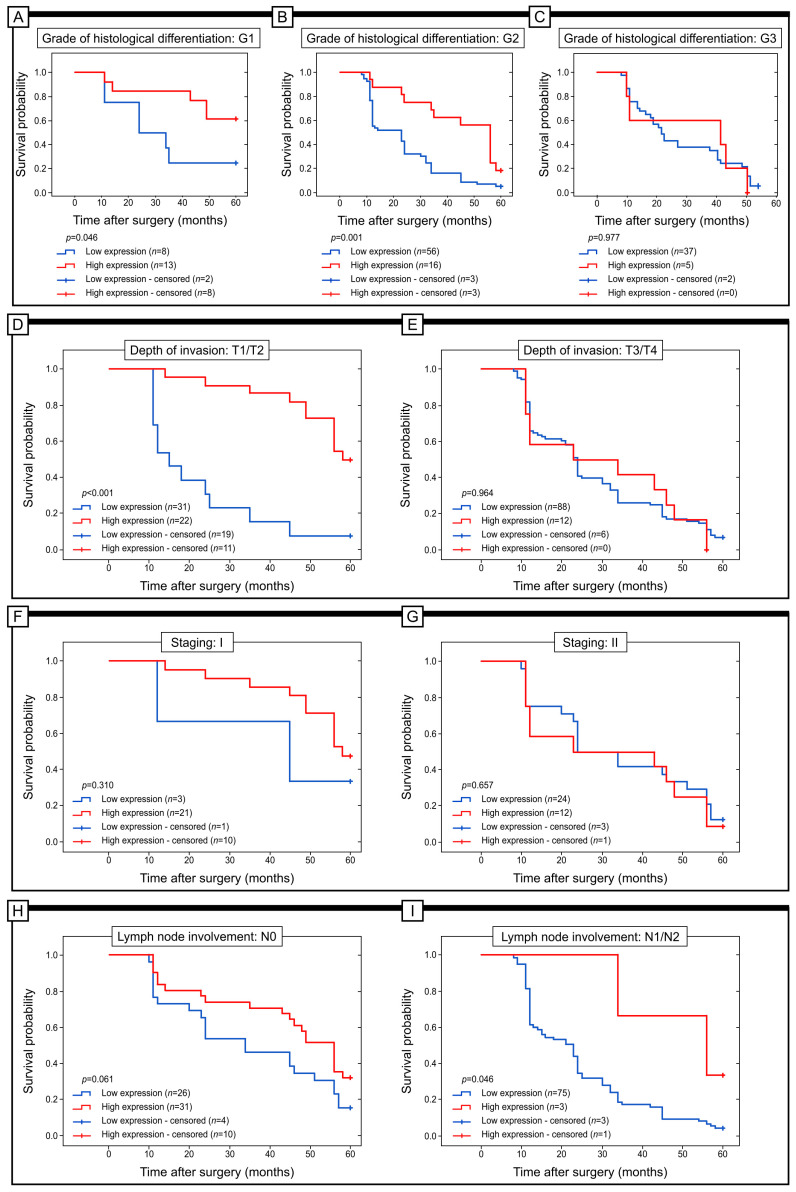
Kaplan–Meier curves showing the survival probability of patients stratified according to the degree of histological differentiation (**A**–**C**); depth of invasion (**D**,**E**); staging (**F**,**G**); lymph node involvement (**H**,**I**) in colon adenocarcinoma samples with high or low Grx1 immunohistochemical expression.

The study examined the relationship between Grx1 immunohistochemical expression and prognosis of patients in different subgroups. Factors such as histological differentiation, depth of invasion, staging, lymph node involvement, and PCNA immunohistochemical expression were taken into account (Figure 3). 

**Figure 4:**
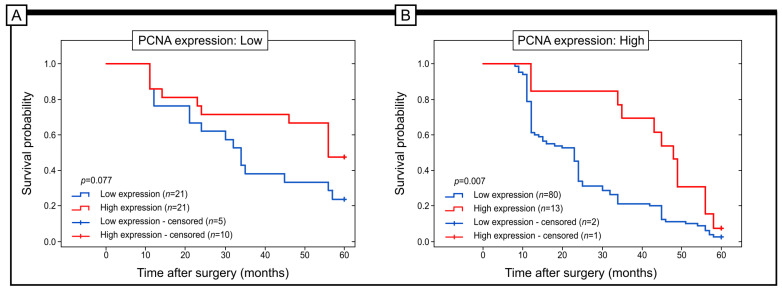
Kaplan–Meier curves showing the probability of survival of patients with high versus low levels of immunohistochemical expression of Grx1 in patients stratified according to the immunohistochemical expression of PCNA in samples of colon adenocarcinoma (**A**,**B**).

The results showed that Grx1 protein expression did not significantly affect the 5-year survival rates of patients in G3 (log-rank test; *p* = 0.977). However, in patients classified as G1 and G2, higher levels of Grxr1 expression were associated with better 5-year survival (log-rank test; *p* = 0.046 and *p* = 0.001, respectively). Similar results were observed in patients with T1/T2 and T3/T4 disease depths. In the T1/T2 group, the high level of Grx1 expression was connected with a better prognosis (log-rank test; p< 0.001). In the T3/T4 group, we did not find statistical significance (log-rank test; *p* = 0.964, respectively). Moreover, we did not find any statistical significance connected with the 5-year survival and Grx1 expression when we stratified patients according to the stage of the disease, e.g., stages I, II, III (log-rank test; *p* = 0.310, *p* = 0.657, *p* = 0.637, respectively). The high immunohistochemical expression of Grx1 protein was also related to better survival in patients with high expression of PCNA protein (log-rank test; *p* = 0.007) (Figure 4). 

The results of the study indicate that Grx1 immunoexpression is among prognostic factors associated with a patient’s clinical outcome. However, Grx1 cannot be considered an independent indicator associated with the 5-year survival rate. This was determined through multivariate analysis. Statistical analyses revealed that staging, depth of invasion, and PCNA expression are independent prognostic factors for our cohort of patients (Table 4). 

### 2.4. Immunofluorescence Staining

**Figure 5:**
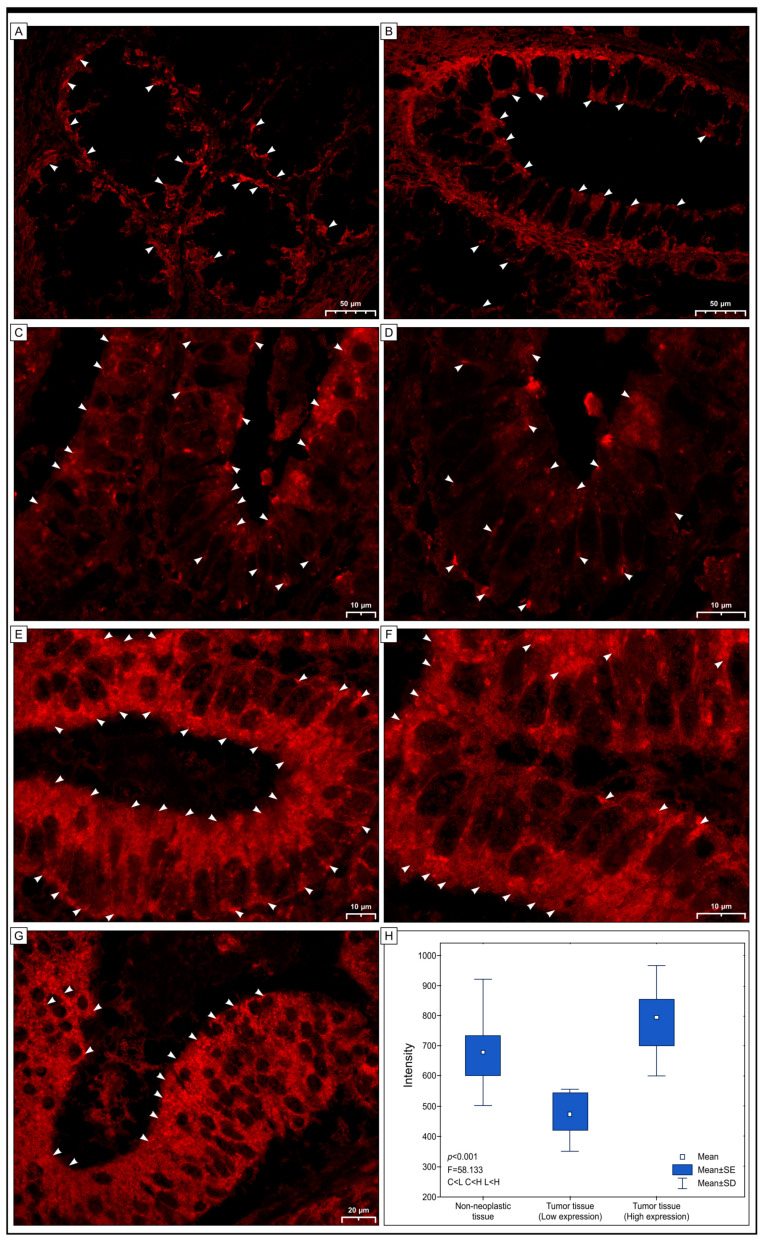
The images below visualize Grx1 protein expression in colon adenocarcinoma (**C**–**G**) and non-cancerous margins (**A**,**B**) through immunofluorescence. A red fluorescent signal of variable intensity, showing the expression of Grx1 in the cytoplasm of colon cells and carcinoma cells (**C**–**G**), is seen in the cells of non-pathological mucosa (**A**,**B**). The fluorescent signals were located in the cytoplasm of the apical regions of several cancer cells, suggesting the detection of Grx1. In contrast, in some cells, the fluorescence was intense throughout the cytoplasm of the cells (arrowheads) or in the nuclei (arrows). ANOVA results (**H**) reveal the difference in intensity of the red signal representing the Grx1 presence between the groups tested; C > L, C < H, L < H. The intensity differences are between groups; C represents colon tissue without pathological alterations, L is adenocarcinoma tissue with low Grx1 expression, and H is adenocarcinoma tissue with high-intensity Grx1 expression.

Our study aimed to investigate the expression of Grx1 in colon adenocarcinomas by using immunofluorescent staining, based on previous studies [36,37,38]. We randomly selected 50 tissue section slides, which were treated with anti-Grx1 antibody and Dako Liquid Permanent Red chromogen (LPR). The selected slides included 10 control samples, 25 samples with low expression identified by immunohistochemistry, and 25 samples with high expression. Although this method served as an additional measure, the results were promising, indicating that the LPR chromogen treatment of anti-Grx1 antibody-stained tissue sections could benefit immunofluorescence studies. To measure the Grx1 expression levels in both normal and cancerous tissues, we used Zeiss Zen 3.4 (blue edition) software. We observed fluorescent signals of varying intensities in both non-cancerous and cancerous mucosa cells. In colon samples without pathological changes, the signal was very intense. A similar signal, but with a different intensity, was detected in cancer cells. It was found in the apical cytoplasmic regions, while in others, strong fluorescence was observed throughout the cytoplasm of the cells and in nuclei (Figure 5).

### 2.5. Intracellular Localization of Grx1 Protein Using the Method of Immunogold Labeling with the Use of Transmission Electron Microscopy (TEM)

**Figure 6:**
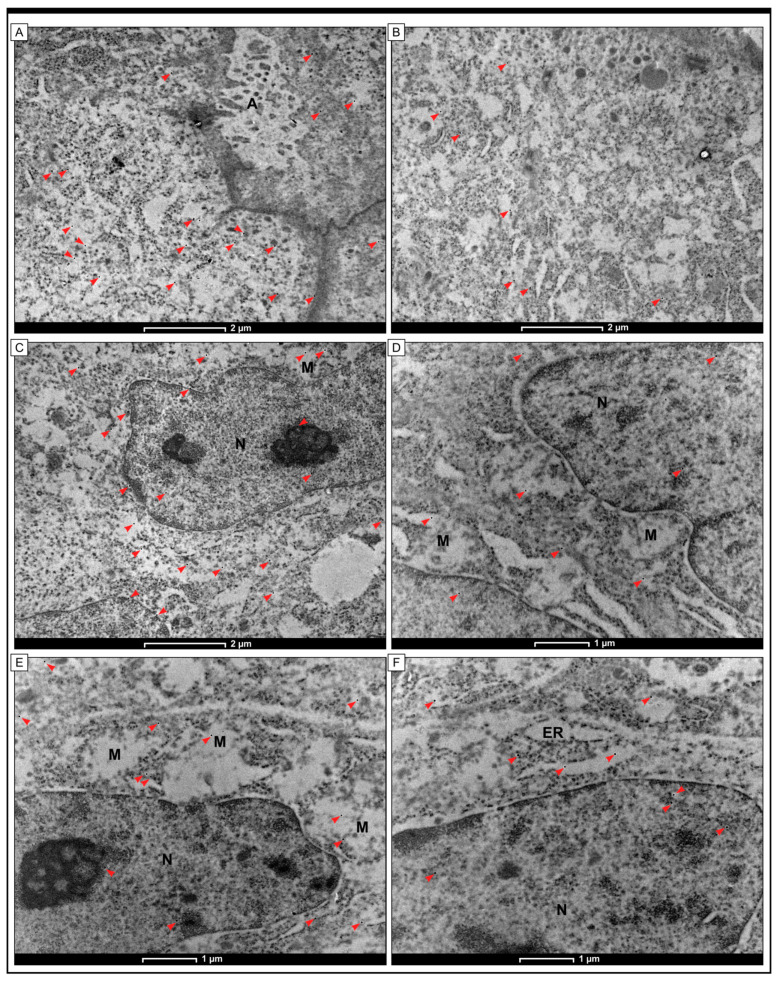
Immunogold labeling was used to detect the presence of Grx1 protein in cells of colon adenocarcinoma tissue at the transmission electron microscopy (TEM) level. The study revealed small electron-dense granules (red arrowheads) in both non-pathological colon mucosae (**A**,**B**) and colon adenocarcinoma samples (**C**–**F**). In non-pathological samples, electron-dense granules indicating the presence of Grx1 were located in the cytoplasm and in the plasma membrane near adherent junctions. Conversely, in cancer cells, black granules were found in the cytoplasm within the mitochondria and cisterns of the endoplasmic reticulum. In some cells, the electron-dense granules were also observed in the nucleus (N). The scale bar for (**A**–**C**) is 2 µm, while for (**D**–**F**), it is 1 µm.

The study utilized an immunogold labeling method to demonstrate the presence of the Grx1 protein in colon adenocarcinoma cells. The results showed that black granules, indicative of the presence of Grx1, were present in both the cytoplasm and nucleus of cancer cells. Furthermore, electron-dense granules were detected in the mitochondria and cisterns of the rough endoplasmic reticulum. In colonocytes from non-pathological samples, scattered black granules were observed in the apical regions of the cytoplasm and in the vicinity of the plasma membrane. The study also found the presence of Grx1 in the mitochondria and cisterns of the endoplasmic reticulum (Figure 6).

### 2.6. GRX1 Gene Expression in Colorectal Cancer Cell Lines

For investigating the expression of the relative gene expression (RQ) of *GRX1*geneunder in vitro conditions, various colorectal cancer cell lines, including SW 1116 (Duke A), LS 174T (Duke B), and HCA-2 (Duke C), were selected for in vitro testing. The CCD 841 CoN line was used as a calibrator in the comparative threshold cycle (Ct) method 2^−∆∆Ct^. A qRT-PCR analysis revealed that the highest levels of *GRX1*gene expression were detected in the LS 174 T cell line (Duke A; Stage I). The lowest level of GRX1 mRNA was detected in the HCA-2 cell line. 

**Figure 7:**
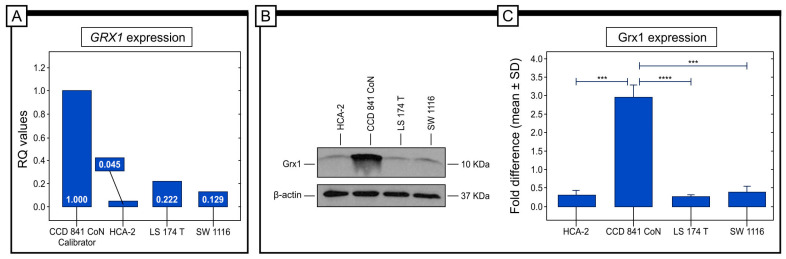
The objective of the study was to analyze the expression of the GRX1gene and protein in different types of colorectal cancer cell lines. The study found Relative Quantification (RQ) gene expression level of *GRX1* in various colorectal cancer cell lines (**A**). The protein expression levels of Grx1 were determined through the Western blot method. It was discovered that the highest level of Grx1 protein expression was in the SW1116 cell line, while the lowest was in the HCA-2 cell line (**B**,**C**). The statistical significance of *p*-values is represented as follows: *** *p* < 0.001; **** *p* < 0.0001.

The protein expression levels in the cancer cell lines were assessed using the Western blot technique in vitro. The results showed that among the cancer cell lines, the SW 1116 cell line had the highest level of Grx1 protein expression. On the other hand, LS 174T and HCA-2 cells had similar levels of expression. It should be mentioned that the highest level of Grx1 expression was detected in the control cell line. Statistical analyses revealed significant differences in Grx1 protein expression between the CCD 841 CoN and HCA-2 cell lines, between CCD 841 CoN and LS 174 T, and between the CCD 841 and SW 1116 cell lines (refer to Figure 7).

### 2.7. Serum Level of Grx1 in Patients

**Figure 8:**
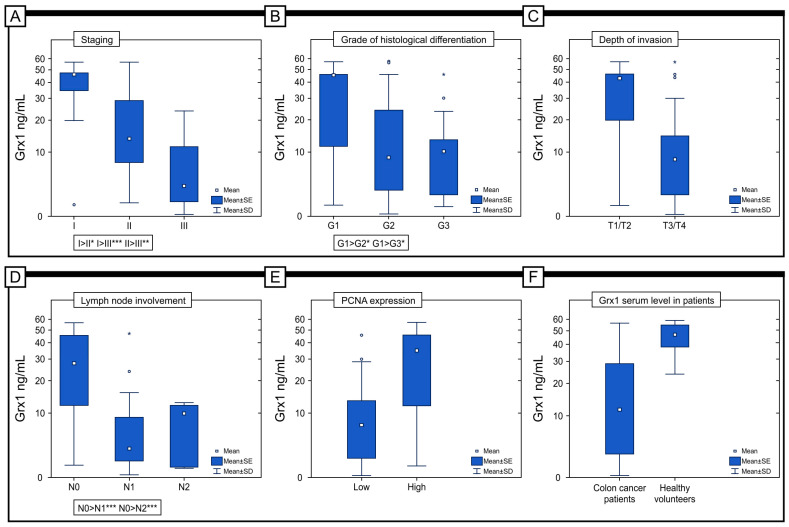
Comparison of serum Grx1 concentrations in colon adenocarcinoma patients according to the staging (**A**), grade of histological differentiation (**B**), depth of invasion (**C**), lymph node involvement (**D**), and immunohistochemical expression of PCNA protein (**E**). (**F**) The concentration of serum level in colon adenocarcinoma patients and healthy volunteers. * *p*< 0.05, ** *p*< 0.01, *** *p* < 0.001.

The concentration of Grx1 in the serum of patients with colon adenocarcinoma (M = 17.76 ng/mL; Me = 11.61 ng/mL) was significantly lower than in the serum of healthy volunteers (M = 45.43 ng/mL; Me = 47 ng/mL) (*p* < 0.001) (Figure 8F). In addition, there was a statistically significant difference in the level of Grx1 in serum between patients with different stages of the disease, with the highest level of Grx1 detected in patients with stage I disease (M = 40.69 ng/mL; Me = 45.66 ng/mL) and the lowest level detected in patients with stage III disease (M = 6.21 ng/mL; Me = 3.55 ng/mL) (*p* < 0.001). In the case of histological differentiation, the highest level was found in patients with G1 (M = 31.82 ng/mL; Me = 45.55 ng/mL), and the lowest level was found in the G3 subject (M = 11.65 ng/mL; Me = 10.12 ng/mL) (*p* = 0.016). Importantly, significantly higher levels were found in patients with T1/T2 (M = 34.86 versus M = 12.03 ng/mL) (*p* < 0.001) and N0 disease (M = 28.13 versus 7.43 versus 7.57 ng/mL) (*p* < 0.001), respectively, for the criteria of depth of invasion (T) and lymph node involvement (N). In our study, we also tried to answer the question of whether the expression of Grx1 in the tumor tissue correlated with the Grx1 content in the serum of patients. The study showed that a higher serum level of Grx1 characterizes patients with a high expression of Grx1 in the colon adenocarcinoma samples (M = 31.81 versus M = 10.29 ng/mL) (*p* < 0.001). 

**Figure 9:**
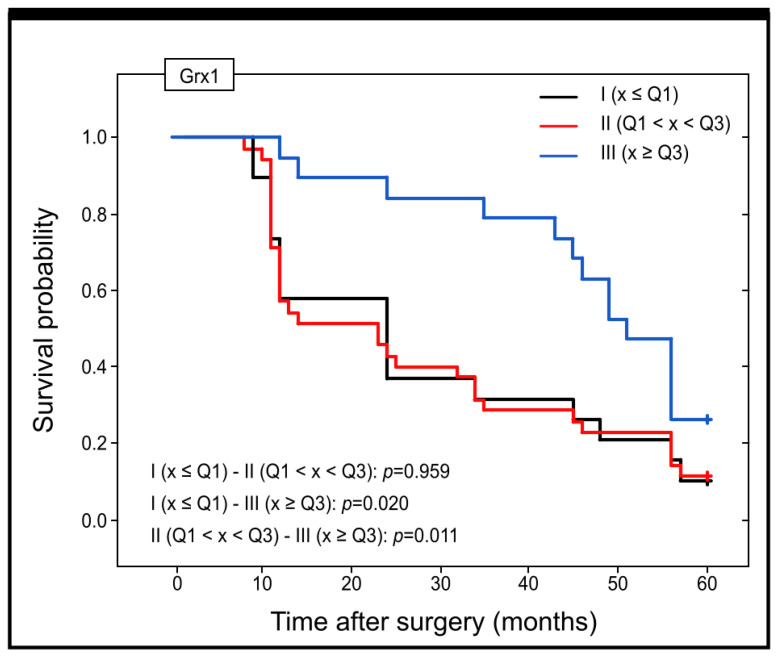
Kaplan–Meier curves showing the survival probability and its changes within the 60 months of the follow-up period for patients with different levels of Grx1 concentration in serum of colon adenocarcinoma patients.

Taking into account the concentration of Grx1 in the serum of patients and its prognostic significance, it should be pointed out that there was a statistically significant difference in estimated survival time between patients with low levels of Grx1 in blood serum (I group) and those with high levels of this protein (III group) (OS Me = 24 versus OS Me = 51) (*p* = 0.020). A significant difference was also detected between patients in groups II and III (OS Me = 23 versus OS Me = 51) (*p* = 0.011) (Figure 9).

## 3. Discussion

The maintenance of intracellular redox balance relies heavily on enzymatic systems, with GSH playing a key role in not only antioxidant defense systems but also in various metabolic processes. Elevated GSH levels have been observed in different types of tumors, making neoplastic tissues more resistant to chemotherapy [5,39,40,41]. In some tumor cells, a high GSH content is typically associated with increased levels of GSH-related enzymes, such as γ-glutamylcysteine ligase (GCL) and γ-glutamyl-transpeptidase (GGT) activities, as well as higher expression of GSH-transporting export pumps [42,43]. Therefore, the GSH system and enzymes associated with its metabolism have attracted attention as promising prognostic tools and targets for medical intervention against cancer progression and chemoresistance [5,39,40,44,45]. Changes in the activities of antioxidant enzymes observed in colorectal cancer patients could represent an index of oxidative stress and potential biomarkers associated with the patient’s prognosis. For example, an increase in SOD activity that transforms superoxide to H_2_O_2_ and a decrease in CAT and GR, which cannot counteract the overproduction of H_2_O_2_, lead to the accumulation of deleterious H_2_O_2_, shifting the redox balance [46,47]. Several studies have shown that the glutathione consumption and recycling system, GPX and GR, are reduced in tumor tissue compared to non-tumor adjacent tissue in colorectal cancer samples. This decrease in the glutathione consumption and recycling system leads to the accumulation of H_2_O_2_, which can cause oxidative stress and enhance the initiation and progression of cancer [48,49,50]. Acevedo-Leon found significant differences in the levels of both GSH and GSSG in the CRC group compared to the healthy control group. GSH levels decreased by over 50%, while GSSG levels increased by over 140%, resulting in a pronounced elevation in the GSSG/GSH% serum ratio in CRC patients, indicating a significant alteration in the redox state of these CRC patients in the direction of oxidation [51]. Like other investigations, a correlation analysis showed a relationship between levels of glutathione and inflammatory markers [52,53,54,55,56]. Ki et al. demonstrated that the mRNA and protein expression of GSH, the catalytic subunit of GCL (GCLC) and GSS, were significantly increased in the following five colon cancer cell lines: Caco 2, SNU 407, SNU 1033, HCT 116, and HT 29, when compared to the normal colon cell line FHC. GSH expression levels were also found to be elevated in tumor tissue when compared to adjacent normal tissue. The results of immunohistochemical studies showed that GCLC and GSS were higher in colorectal cancer tissue than in normal mucosa. Since GSH and GSH-metabolizing enzymes are found at increased levels in colon tumors, they could be useful clinically as biomarkers for colon cancer and/or targets for anti-cancer therapy [57].

In the context of Grx1 activity, it should be mentioned that silencing of the *GRX1* gene caused a significant change in GSH/GSSG ratio, leading to ROS hyperaccumulation; in turn, overexpression of *GRX1* reduced cellular ROS levels [29]. In addition, *GRX1* knockdown increased the S- glutathionylation of DJ-1 and HSP60 proteins, leading to a decrease in mitochondrial respiratory capacity and ATP production. The silencing of *GRX1* activated p53 signaling, which inhibited the G1-S transition via CDK4, leading to a cell cycle arrest in the G1 phase and cell ageing [58]. The exposure of oral squamous cell carcinoma cells (CAL27) to Interleukin-1 beta (IL-1β) activity led to an increase in the levels of Grx1 protein. This increase, in turn, caused a reduction in the levels of ROS and made CAL27 cells more invasive and mobile [59]. After subcutaneous implantation of melanoma cells, a mouse model with endothelial cell-specific overexpression of *GRX1* showed increased tumor growth, but angiogenesis was suppressed [60]. In the tumor model, STAT5 triggers Bcr-Abl mutation, which inhibits *GRX1* gene expression, leading to an increase in ROS levels and a decrease in cell viability in chronic myeloid leukemia [61]. 

Our study is the first to address the complex issue of the clinical and prognostic significance of the Grx1 protein in colon cancer, particularly adenocarcinoma. At this stage of our research, we have focused mainly on patients with stages I, II, and III disease, i.e., patients diagnosed with a primary tumor in the colon (I, II) and those with cancer metastases in the regional lymph nodes (III). Stage IV patients are a very complex and heterogeneous group that requires special and separate analysis, and we have deliberately excluded them from our group at this stage of our research. However, we intend to also examine the expression of Grx1 protein in this group of patients in the future.

We have shown that high immunohistochemical expression of Grx1 protein was detected mainly in lesion-free samples and patients with stage I disease. The low expression of Grx1 in patients with stages II and III disease may be due to Grx1 inhibition associated with increased levels of oxidative stress. This is because Grx1 has additional structural cysteine residues that are sensitive to oxidative stress, and when Grx1 activity is inhibited, a second Grx, Grx2, is activated. The reason why Grx2 is less sensitive to oxidative stress is its ability to form Fe-S clusters [62,63]. The high expression of Grx1 in samples without any pathological changes is associated with its activity as a major GSH-related protein associated with deglutathionylation and the fact that the gastrointestinal tract and the large bowel are particularly exposed to oxidative stress [51]. The high expression of Grx1 in colon tissue may prevent the continuous formation of protein-bound GSH (PSSG), which can lead to significant changes in the structure, function, and activity of many proteins involved in cell proliferation, cell death, and metabolism [64]. For example, the actin rearrangement is linked to its S-glutathionylation. This, in turn, affects the EMT process, which is necessary for the acquisition of a mesenchymal phenotype capable of migration and invasion [33]. Although the cellular environment and GSH can remove protein S-glutathionylation without enzymatic catalysis, the process is slow [65]. For example, Glrx-deficient mice are not lethal, but their tissues have significantly higher levels of protein S-glutathionylation under oxidative stress [66,67]. It seems, therefore, that the high expression of Grx1 protein in the samples without any pathological changes and early stages of colorectal carcinogenesis may play a protecting role [68]. It is significant to note that the studies analyzing Grx1 levels in patients’ blood confirmed these results. In colon cancer patients, the serum’s highest levels were found in stage I patients, which was reflected in the survival curves. A higher level of Grx1 in the serum has been associated with a more favorable outcome. This suggests that Grx1 protein may be a promising approach to colon adenocarcinoma, and its low level of expression could potentially be used as a biomarker to identify patients with a more aggressive form of this cancer. 

Our research findings indicate a significant correlation between Grx1 immunohistochemical expression and various factors related to tumor progression, such as tumor histological grade, depth of invasion, angioinvasion, staging, lymph node involvement, and PCNA immunohistochemical expression. We found that patients with G1 and G2 grades had a higher level of Grx1 compared to those with G3. In the context of other clinical parameters, including depth of invasion and regional lymph node involvement in patients described as T3/T4 and N1/N2, a low level of Grx1 protein was described—88% and 96%, respectively. The analysis of clinical factors showed that one of the factors that can be considered independent in the diagnosis of colon adenocarcinoma in our cohort of patients is the stage. Our study showed that the highest level of expression is characteristic for patients with stage I disease, whereas the vast majority of patients with stage III disease have low levels of this protein. These results were supported by in vitro analysis conducted on colorectal cancer cell lines that corresponded to stages I, II, and III of colorectal cancer, using qRT-PCR and Western blot. In addition, there was a statistically significant difference in the level of Grx1 in serum between patients with different stages of the disease, with the highest level of Grx1 detected in patients with stage I disease and the lowest level detected in patients with stage III disease (*p* < 0.001). Taking into account the concentration of Grx1 in the serum of patients and its prognostic significance, it should be pointed out that there was a statistically significant difference in estimated survival time between patients with low levels of Grx1 in blood serum (I group) and those with high levels of this protein (III group) (OS Me = 24 versus OS Me = 51) (*p* = 0.020).

In the context of the planned future research, it seems to be important to know the intracellular localization of the Grx1 protein. The results of our study showed that Grx1 protein was present in the cytoplasm of cancerous cells. The black electron-dense granules that indicated the presence of Grx1 protein were associated with the cell membrane, endoplasmic reticulum membranes, and mitochondria. In some cells, Grx1 was also found in the nuclei. The distinct localization of Grx1 within different compartments of cells may be linked to its various functions. While Grx1’s primary role is to maintain cellular redox balance, it is also involved in pro-inflammatory and anti-apoptotic activities. These abilities may be specifically related to the nuclear isoform of Grx1. 

Unfortunately, there is little published data regarding Grx1 expression in cancer. As revealed by the studies, Grxs proteins are widely expressed in human lung tissues, e.g., healthy lung, parenchymal sarcoidosis, extrinsic allergic alveolitis, and simple interstitial pneumonia and lung cell lines [69,70,71,72]. Significantly, in lung adenocarcinoma and squamous cell carcinoma, the expression of *GRX1* is decreased [70,71]. In contrast, Nakamura et al. revealed the association of *GRX1* with the malignancy of pancreatic ductal carcinoma where the expression of *GRX1* was higher than that in pancreatic cystadenocarcinoma or normal pancreas tissue [73]. Moreover, the expression of *GRX1* in cis-diamminedichloroplatinum (CDDP)-resistant subclones of HeLa cells was also higher in comparison to parental HeLa cells [74]. Elevated expression levels of the Grx1 protein are noted in hepatocellular cancer patients, and, in this case, it may act as a prognostic factor [75]. Grx1 exerts contradictory influences on HepG2 cells. In this case, it is required for proliferation but also contributes to the antiproliferative effect of NO, associated with Akt1 redox changes [75]. González et al. demonstrated that the antiproliferative effect of NO is hampered by Trx1 and Grx1 and support the strategy of weakening the thiolic antioxidant defenses during designing new antitumoral therapies [76]. Interestingly, Brancaccio et al. demonstrated that in HepG2 cells, the other enzymes associated with redox balance are overexpressed, including GGT. This indicates that in normal cells such as liver cells, GGT physiological levels may be involved in the regulation of the cellular redox balance, levels of GSH, amino acid metabolism, and regulated autophagy. On the other hand, GGT-overexpressing cell lines may be distinguished by the modification of autophagic machinery, favoring the survival and proliferation of cancer cells [10,77].

It is widely recognized that factors such as nodal status (pN) and depth of primary tumor infiltration (pT) are important prognostic factors for local recurrence and distant metastasis in cancer patients [78]. Our study found that most patients with the T3/T4 depth of tumor invasion and N1/N2 nodal status had low levels of Grx1 expression. As the disease progresses, it appears that the expression of Grx1 decreases. Moreover, our study found that the expression of Grx1 significantly distinguished colon adenocarcinoma patients from healthy controls, not only in the case of expression within the cancerous tissues but also in the case of serum Grx1 analysis. At this moment, the only reliable test to determine the cancer stage is the histopathological analysis of the resected tumor and surrounding tissues. However, discovering new biomarkers that can be assessed in serum samples may be helpful for the pre-operative diagnosis and non-invasive evaluation of cancer progression. This could help in choosing appropriate treatment and improving the survival rate of colorectal cancer patients.

## 4. Conclusions and Limitations of the Study

Our research examined the expression of Grx1 protein in the colon adenocarcinoma tissue of patients from European populations (Polish) who are in stages I, II, and III of the disease. We used immunohistochemical and immunofluorescence techniques for this purpose. Out of the samples we studied, 34 cases of colorectal adenocarcinoma (25.19%) had high levels of Grx1 protein expression, while 101 cases (74.81%) had low levels of Grx1 protein expression. Our research showed that the high expression of Grx1 was detected in tissue samples without any pathological changes and in patients with stage I of the disease. We showed that Grx1 expression levels decreased gradually with the increase in the depth of invasion (pT) and nodal status (N). Moreover, we observed a trend indicating that patients with a higher expression of the Grx1 protein have a significantly better 5-year survival rate compared to those with lower levels of this protein (log-rank, *p* < 0.001). The expression of Grx1 is among the prognostic factors in colon adenocarcinoma patients (univariate analysis), but it should not be regarded as an independent prognostic factor in our cohort of patients (multivariate analysis). The prognostic activity should be regarded together with such clinical parameters as depth of invasion (T status) or PCNA expression. In the context of Grx1 analysis in the serum, the highest levels of Grx1 protein are also found in stage I patients, which is extremely significant for clinical diagnosis. The identification of new biomarkers through serum samples can facilitate the non-invasive monitoring of cancer progression, allowing for the selection of appropriate treatment and improving the survival rate of CRC patients.

Our study has some limitations, such as the limited size of the cohort studied, which may have introduced selection bias into the study. Therefore, future studies should be conducted to increase the sample size and to understand the mechanism of Grx1 activity through in vitro molecular experiments. Nonetheless, the study meticulously selected a group of patients without any accompanying diseases, and this marks the beginning of more clinical trials assessing the diagnostic utility of redox biomarkers in a larger population of colorectal cancer patients.

## 5. Materials and Methods

### 5.1. Samples from Tumors and Patients

Colon tissue samples were collected from individuals with colon adenocarcinoma confirmed through histopathological examination during colon resection at Jaworzno Municipal Hospital from January 2014 to December 2017. The study excluded patients who had undergone preoperative radiotherapy or chemotherapy, had severe medical conditions or distant metastases, had inflammatory bowel disease, had tumor recurrence or had a histopathological subtype other than adenocarcinoma. We followed a standardized protocol and obtained histopathological sections from each surgical specimen, including tumor fragments and adjacent tissue without tumor abnormalities. The specimens were preserved in formalin and then embedded in paraffin blocks. Later, the blocks were sectioned and stained with H&E to diagnose histopathology. We also examined the marginal tissue sections, and if any cancer cells were detected, we excluded the material from the study. The patients were followed up for 5 years to assess the prognostic significance of the Grx1 protein.

### 5.2. Immunohistochemical and Immunofluorescence Staining

Colon adenocarcinoma specimens and resected margins were processed in formalin and embedded in paraffin blocks. From these blocks, 4 µm thick sections were cut and placed on polysine slides. Sections were incubated with glutaredoxin 1 and PCNA antibodies (GeneTex. Irvine, CA, USA Polyclonal Antibodies. Cat. No. GTX54140 and GTX100539, respectively) at final dilutions of 1:700 and 1:600. Bright-Vision and Permanent AP Red Chromogen were used to visualize protein expression. Mayer’s hematoxylin was used to counterstain nuclei. We followed the immunoreactive score used in previous publications [79,80] to analyze the results of immunohistochemical staining. Both intensity and number of cells with positive reactions were used in the score. The intensity was scored in the following way: 0 for no signal, 1 for weak, 2 for moderate, and 3 for strong staining. The frequency of positive cells was assessed semiquantitatively through the evaluation of the entire section, and each sample was scored on a scale of 0 to 4:0 for negative, 1 for positive staining in 10–25% of cells, 2 for 26–50% of cells, 3 for 51–75% of cells, and 4 for 76–100% of cells. We then calculated a total score of 0–12 and graded it as follows: I for scores 0–1, II for scores 2–4, III for scores 5–8, and IV for scores 9–12. Score I was regarded as negative and scores II, III, and IV as positive. Scores I and II represented low expression (no or weak staining), and scores III and IV represented high expression (strong staining). Sections were treated with anti-Grx1 antibody and Dako Liquid Permanent Red (LPR) for immunofluorescence. Fluorescence of Grx1 protein was observed using a confocal fluorescence microscope (Zeiss LSM 980 with Airscan 2; Zeiss; Germany) with excitation at 592 nm and emission between 574 and 735 nm using TexRed filter kits. Zeiss Zen 3.4 (blue edition) version 3.4.91.00000 (Zeiss; Germany) was used to measure the intensity of Grx1 expression in both non-neoplastic and tumor tissue.

### 5.3. Immunogold Electron Microscopy

In this study, the samples were fixed by immersing them in a solution of 4% paraformaldehyde in 0.1 M phosphate-buffered saline (PBS) at room temperature for two hours. After that, they were rinsed several times in PBS. Next, the specimens were dehydrated in a series of graded ethanol and infiltrated for 30 min on ice in a mixture of 2 parts ethanol to 1 part LR White and a mixture of 1 part ethanol to 2 parts LR White. Following the infiltration with pure LR White, ultrathin sections (70 nm) were cut using an RMC Boeckeler Power Tomo PC ultramicrotome equipped with a 45° diamond blade (Diatom AG, Biel, Switzerland). The ultrasections were then immunolabeled and placed onto nickel grids coated with Formvar. Beforehand, the sections on the grids were pre-incubated for 30 min by floating on drops of 50 mM NH4 Cl in PBS, followed by 30 min of blocking on drops of 1% BSA in PBS. The grids were treated overnight (16–18 h) at a temperature of 4 °C with a 1:20 dilution of primary anti-Grx3 antibody in BSA. The bound antibodies were localized by incubating the sections with immunogold conjugated goat anti-mouse IgG 15 nm (BBInternational BBI Solutions, Sittingbourne, UK) diluted 1:100 for 1 h. Afterwards, the grids were rinsed with PBS drops (five changes, 5 min each) and water (three changes, 3 min each) before staining with 0.5% aqueous uranyl acetate. The main antibody was not included in the control group. After preparation, the grids were air-dried and examined under a TECNAI 12 G2 Spirit Bio Twin FEI Company transmission electron microscope at 120 kV. The images were captured using a Morada CCD camera (Gatan RIO 9, Pleasanton, CA, USA).

### 5.4. Colorectal Cancer Cell Lines

For the experiments, three different colorectal cancer cell lines were used—HCA-2 (Duke C), LS 174T (Duke B), and SW 1116 (Duke A), along with a normal epithelial cell line known as CCD 841 CoN. All the cell lines were provided by ATCC (American Type Culture Collection ATCC^®^, Old Town Manassas, VA, USA). To promote optimal cell growth, we utilized specific culture media for each cell line. For CCD 841CoN and LS 174T cell lines, we used Eagle’s minimum essential medium (EMEM) (ATCC 30-2003), while Dulbecco’s modified Eagle’s medium/Nutrient Mixture F-12 Ham (DMEM) from Sigma-Aldrich D8437 was used for the SW 1116 and HCA-2 cell lines. Additionally, both media were supplemented with 10% fetal bovine serum ((FBS), ATCC 30-2020), and 1% penicillin-streptomycin-neomycin stabilized solution (Sigma-Aldrich P4083) for optimal growth conditions.

### 5.5. GRX1gene Expression in Colorectal Cancer Cell Lines

RNA was isolated using the RNA Isolation Kit (BioVendor, Brno, Czech Republic) and analyzed using spectrophotometry in a Biochrom WPA Biowave DNA UV/Vis Spectrophotometer (Biochrom, Business Park, Building 1020, 1010 Cambourne Rd, Cambourne, Cambridge CB23 6DW, UK). As part of the investigation, we proceeded to synthesize complementary DNA (cDNA) from the isolated RNA. To achieve this, we carried out a reverse transcription reaction using a High Capacity cDNA Reverse Transcription Kit with RNase Inhibitor from Applied Biosystems (ThermoFisher Scientific, Waltham, MA, USA), following the manufacturer’s instructions. The next step involved the analysis of relative gene expression through Quantitative Reverse Transcription Polymerase Chain Reaction (qRT-PCR) using specific TaqMan^®^ Gene Expression Assays, also from Applied Biosystems (USA). For the GLRX1 gene (Hs00829752_g1), Q-PCR was conducted, with the glyceraldehyde-3-phosphate dehydrogenase gene (GAPDH, Hs03929097_g1) used as an endogenous control. The reference sample, or calibrator, was a human cell line CCD 841 CoN. The qRT-PCR was performed in a 20 µL volume, where 1 µL of cDNA, 10 µL of TaqMan^®^ Gene Expression Master Mix (from Applied Biosystems, USA), 1 µL of the primer and probe mix (TaqMan^®^ Gene Expression Assays), and 8 µL of H_2_O (from EURx, Gdańsk, Poland) were used. The genes were subjected to a thermal cycle consisting of an initial denaturation step of 95 °C for 10 min, followed by 40 cycles of 95 °C for 15 s and 60 °C for 1 min. The assays were performed using the QuantStudio 5 Real-Time PCR System (Applied Biosystems, USA). For the initial denaturation step, the samples were heated at 95 °C for 10 min, followed by 40 cycles of heating at 95 °C for 15 s, and then cooling at 60 °C for 1 min. The assays were conducted using the QuantStudio 5 Real-Time PCR System made by Applied Biosystems, USA. 

In the study that utilized the Western blot method, the cells were first rinsed once in PBS and then lysed in TLB. Then, 10 µg of protein was separated using SDS-PAGE (15%) for 1.5 h at 150 V and transferred to a nitrocellulose membrane via wet blotting in transfer buffer. The cells were initially rinsed once in PBS and then lysed in TLB. Next, 10 µg of protein was separated via SDS-PAGE (15%) for 1.5 h at 150 V and transferred to a nitrocellulose membrane using wet blotting in transfer buffer. For primary antibody incubation, Primary Antibody Diluent (SuperSignal Western blot Enhancer, Thermo Scientific ThermoFisher Scientific, Waltham, MA, USA, 46641) was used, and the incubation was carried out overnight at 4 °C. Following washing in TBST, the membrane was incubated with HRP-conjugated secondary antibody in 5% skim milk/TBST for 1 h at room temperature. The following antibodies were used for Western blotting: rabbit anti-Glutaredoxin 1 (GeneTex, Irvine, CA, USA GTX54140; 1:1000), mouse anti-β-Actin (R&D SYSTEM, MAB8929; 1:1000), HRP-coupled mouse anti-rabbit IgG (Invitrogen, Waltham, MA; 31464; 1:25,000), and goat anti-mouse IgG (Invitrogen, A16078; 1:25,000).

### 5.6. Serum Level of Grx1 in Colon Adenocarcinoma Patients

The study group consisted of 73 colon adenocarcinoma patients (40 males and 33 females, aged 36–89 years) and 20 healthy volunteers with a negative history of inflammatory diseases and cancers as the control group. Blood samples were taken from colon adenocarcinoma patients before the treatment and frozen at −80 °C. The serum level of Grx1 was assessed with the enzyme-linked immunosorbent assay (ELISA) according to the manufacturers’ instructions (SEG981Hu 96 Tests Enzyme-linked Immunosorbent Assay Kit for Glutaredoxin (GLRX); Cloud- Clone Corp. Wuhan; China). For statistical analysis, all subjects were divided into three groups according to the quartile range of Grx1 serum levels in the total group (Table 5).

### 5.7. Statistical Analysis

In this study, we analyzed the relationship between Grx1 immunohistochemical expression and relevant clinical parameters using Statistica 9.1 software developed by StatSoft in Krakow, Poland. To evaluate all numerical variables, we used the statistical measures of median and range. We assessed the relative characteristics of the groups studied using both the chi-squared test (Ch^2^ test) and Yates’ chi-squared test (Chi^2^_Yatesa_ test). In the case of the correlation between Grx1 and PCNA treated as dependent variables, we used the McNemara test. The study assessed the relationship between Grx1 expression and patient survival using Kaplan–Meier analysis and log-rank test. Statistical significance was set at *p* < 0.05. A similar test was used to assess the probability of colon adenocarcinoma patients in relation to Grx1 serum level. For the analysis of the relationship between clinical parameters and Grx1 serum level, the Wilcoxon paired *t*-test was used.

## Figures and Tables

**Table 1:** Characteristics of patients included in the study (n = 135).

		N (Number of Cases)	%
Gender	Females	70	51.85
	Males	65	48.15
Age [years]	≤60 years	54	40.00
	61–75 years	43	31.85
	>75 years	38	28.15
	M ± SD	64.10 ± 13.47	
	Me [Q1–Q3]	64 [Age range: 55–77]	
	Min–Max	33–89	
Grade of histological differentiation	G1	21	15.55
	G2	72	53.33
	G3	42	31.11
Depth of invasion	T1	14	10.37
	T2	21	15.56
	T3	79	58.52
	T4	21	15.56
Regional lymph node involvement	N0	57	42.22
	N1	45	33.33
	N2	33	24.45
Location of tumor	Proximal	72	53.33
	Distal	63	46.67
Angioinvasion	No	29	21.48
	Yes	106	78.52
PCNA immunohistochemical expression	Low	42	31.11
	High	93	68.89
Staging	I	24	17.78
	II	36	26.67
	III	75	55.55

**Table 2:** Clinicopathological characteristics correlated with Grx1 protein expression in colon adenocarcinoma patients.

		The Immunoexpression Level of Grx1				
		Low		High		*p*-Value
Age [Years]	≤60 years	39	(72.22%)	15	(27.78%)	*p* = 0.764
	61–75 years	32	(74.42%)	11	(25.58%)	
	>75 years	30	(78.95%)	8	(21.05%)	
Gender	Females	52	(74.29%)	18	(25.71%)	*p* = 0.883
	Males	49	(75.38%)	16	(24.62%)	
Grade of histological differentiation	G1	8	(38.1%)	13	(61.9%)	*p* < 0.001
	G2	56	(77.78%)	16	(22.22%)	
	G3	37	(88.1%)	5	(11.9%)	
Depth of invasion	T1/T2	13	(37.14%)	22	(62.86%)	*p* < 0.001
	T3/T4	88	(88,00%)	12	(12,00%)	
Regional lymph node involvement	N0	26	(45.61%)	31	(54.39%)	*p* < 0.001
	N1/N2	75	(96.15%)	3	(3.85%)	
Location of tumor	Proximal	52	(72.22%)	20	(27.78%)	*p* = 0.458
	Distal	49	(77.78%)	14	(22.22%)	
Angioinvasion	No	16	(55.17%)	13	(44.83%)	*p* = 0.012
	Yes	85	(80.19%)	21	(19.81%)	
PCNA immunohistochemical expression	Low	21	(50,00%)	21	(50,00%)	*p* < 0.001
	High	80	(86.02%)	13	(13.98%)	
Staging	I	3	(12.5%)	21	(87.5%)	*p* < 0.001
	II	24	(66.67%)	12	(33.33%)	
	III	74	(98.67%)	1	(1.33%)	

**Table 3:** An analysis of the relationship between Grx1 protein expression and PCNA protein expression.

		The Immunoexpression Level of Grx1				*p*-Value
		Low		HIGH		
PCNA expression	Low	21	(15.56%)	21	(15.56%)	*p* = 0.230
	High	80	(59.26%)	13	(9.63%)	*p* < 0.001

**Table 4:** Cox regression analyses.

Prognostic Parameter	Univariate Analysis			Multivariate Analysis		
	HR	95% CI	*p*-Value	HR	95% CI	*p*-Value
Gender: male (ref: female)	1.033	0.715–1.491	0.864	–	–	–
Age	0.998	0.984–1.012	0.816	–	–	–
Age: 61–75 (ref: <60)	0.923	0.598–1.427	0.719	–	–	–
Age: >75 (ref: <60)	0.974	0.617–1.536	0.910	–	–	–
Staging: II (ref: I)	3.503	1.757–6.986	<0.001	9.864	3.195–30.460	<0.001
Staging: III (ref: I)	5.484	2.855–10.535	<0.001	15.279	2.094–111.483	0.007
G2 (ref: G1)	3.094	1.622–5.902	0.001	1.837	0.697–4.838	0.218
G3 (ref: G1)	2.766	1.409–5.430	0.003	1.281	0.461–3.561	0.635
T3/T4 (ref: T1/T2)	2.429	1.500–3.932	<0.001	0.368	0.174–0.778	0.009
N1 (ref: N0)	2.094	1.351–3.245	0.001	0.809	0.179–3.651	0.783
N2 (re: N0)	2.596	1.588–4.246	<0.001	1.018	0.209–4.964	0.982
Proximal (ref: distal)	0.984	0.680–1.423	0.931	–	–	–
Grx1 expression high (ref: low)	0.373	0.234–0.595	<0.001	1.316	0.666–2.598	0.429
Angioinvasion YES (ref: NO)	2.076	1.235–3.491	0.006	0.811	0.367–1.793	0.605
PCNA expression: high (ref: low)	2.806	1.740–4.527	<0.001	2.064	1.189–3.583	0.010

**Table 5:** Grx1 serum level in colon adenocarcinoma patients.

	Grx1 Serum Level in Colon Adenocarcinoma Patients								
	N	%	M	Me	Min	Max	Q1	Q3	SD
x ≤ Q1	19	26.03	1.56	1.46	0.23	2.88	1.00	2.35	0.68
Q1 < x < Q3	35	47.94	12.02	11.61	3.21	27.76	7.62	14.35	6.39
x ≥ Q3	19	26.03	44.14	45.66	28.73	56.99	34.79	46.97	8.82

## Data Availability

Data are contained within the article.

## References

[1] Bray F., Ferlay J., Soerjomataram I., Siegel R.L., Torre L.A., Jemal A. (2018). Global cancer statistics 2018: GLOBOCAN estimates of incidence and mortality worldwide for 36 cancers in 185 countries. CA Cancer J. Clin..

[2] Siegel R.L., Miller K.D., Goding Sauer A., Fedewa S.A., Butterly L.F., Anderson J.C., Cercek A., Smith R.A., Jemal A. (2020). Colorectal cancer statistics, 2020. CA Cancer J. Clin..

[3] Connell L.C., Mota J.M., Braghiroli M.I., Hoff P.M. (2017). The Rising Incidence of Younger Patients With Colorectal Cancer: Questions About Screening, Biology, and Treatment. Curr. Treat. Options Oncol..

[4] Akimoto N., Ugai T., Zhong R., Hamada T., Fujiyoshi K., Giannakis M., Wu K., Cao Y., Ng K., Ogino S. (2021). Rising incidence of early-onset colorectal cancer—A call to action. Nat. Rev. Clin. Oncol..

[5] Kennedy L., Sandhu J.K., Harper M.E., Cuperlovic-Culf M. (2020). Role of Glutathione in Cancer: From Mechanisms to Therapies. Biomolecules.

[6] Pool-Zobel B., Veeriah S., Böhmer F.D. (2005). Modulation of xenobiotic metabolising enzymes by anticarcinogens—Focus on glutathione S-transferases and their role as targets of dietary chemoprevention in colorectal carcinogenesis. Mutat. Res..

[7] Hanschmann E.M., Godoy J.R., Berndt C., Hudemann C., Lillig C.H. (2013). Thioredoxins, glutaredoxins, and peroxiredoxins--molecular mechanisms and health significance: From cofactors to antioxidants to redox signaling. Antioxid. Redox Signal.

[8] Francisco A., Ronchi J.A., Navarro C.D.C., Figueira T.R., Castilho R.F. (2018). Nicotinamide nucleotide transhydrogenase is required for brain mitochondrial redox balance under hampered energy substrate metabolism and high-fat diet. J. Neurochem..

[9] Castellano I., Merlino A. (2012). γ-Glutamyltranspeptidases: Sequence, structure, biochemical properties, and biotechnological applications. Cell. Mol. Life Sci..

[10] Mitrić A., Castellano I. (2023). Targeting gamma-glutamyl transpeptidase: A pleiotropic enzyme involved in glutathione metabolism and in the control of redox homeostasis. Free Radic. Biol. Med..

[11] Hanigan M.H. (2014). Gamma-glutamyl transpeptidase: Redox regulation and drug resistance. Adv. Cancer Res..

[12] Hanigan M.H., Frierson H.F., Brown J.E., Lovell M.A., Taylor P.T. (1994). Human ovarian tumors express gamma-glutamyl transpeptidase. Cancer Res..

[13] Murata J., Ricciardi-Castagnoli P., Dessous L’Eglise Mange P., Martin F., Juillerat-Jeanneret L. (1997). Microglial cells induce cytotoxic effects toward colon carcinoma cells: Measurement of tumor cytotoxicity with a gamma-glutamyl transpeptidase assay. Int. J. Cancer.

[14] Tsutsumi M., Sakamuro D., Takada A., Zang S.C., Furukawa T., Taniguchi N. (1996). Detection of a unique gamma-glutamyl transpeptidase messenger RNA species closely related to the development of hepatocellular carcinoma in humans: A new candidate for early diagnosis of hepatocellular carcinoma. Hepatology.

[15] Supino R., Mapelli E., Sanfilippo O., Silvestro L. (1992). Biological and enzymatic features of human melanoma clones with different invasive potential. Melanoma Res..

[16] Täger M., Ittenson A., Franke A., Frey A., Gassen H.G., Ansorge S. (1995). gamma-Glutamyl transpeptidase-cellular expression in populations of normal human mononuclear cells and patients suffering from leukemias. Ann. Hematol..

[17] Prezioso J.A., Wang N., Duty L., Bloomer W.D., Gorelik E. (1993). Enhancement of pulmonary metastasis formation and gamma-glutamyltranspeptidase activity in B16 melanoma induced by differentiation in vitro. Clin. Exp. Metastasis.

[18] Obrador E., Carretero J., Ortega A., Medina I., Rodilla V., Pellicer J.A., Estrela J.M. (2002). gamma-Glutamyl transpeptidase overexpression increases metastatic growth of B16 melanoma cells in the mouse liver. Hepatology.

[19] Bard S., Noël P., Chauvin F., Quash G. (1986). gamma-Glutamyltranspeptidase activity in human breast lesions: An unfavourable prognostic sign. Br. J. Cancer.

[20] Traverso N., Ricciarelli R., Nitti M., Marengo B., Furfaro A.L., Pronzato M.A., Marinari U.M., Domenicotti C. (2013). Role of glutathione in cancer progression and chemoresistance. Oxid. Med. Cell. Longev..

[21] Guo L., Chen S., Liu Q., Ren H., Li Y., Pan J., Luo Y., Cai T., Liu R., Chen J. (2020). Glutaredoxin 1 regulates macrophage polarization through mediating glutathionylation of STAT1. Thorac. Cancer.

[22] Xiong Y., Uys J.D., Tew K.D., Townsend D.M. (2011). S-glutathionylation: From molecular mechanisms to health outcomes. Antioxid. Redox Signal.

[23] Chai Y.C., Mieyal J.J. (2023). Glutathione and Glutaredoxin-Key Players in Cellular Redox Homeostasis and Signaling. Antioxidants.

[24] Garg R., Benedetti L.G., Abera M.B., Wang H., Abba M., Kazanietz M.G. (2014). Protein kinase C and cancer: What we know and what we do not. Oncogene.

[25] Steinberg S.F. (2015). Mechanisms for redox-regulation of protein kinase C. Front. Pharmacol..

[26] Velu C.S., Niture S.K., Doneanu C.E., Pattabiraman N., Srivenugopal K.S. (2007). Human p53 is inhibited by glutathionylation of cysteines present in the proximal DNA-binding domain during oxidative stress. Biochemistry.

[27] Zamaraev A.V., Kopeina G.S., Prokhorova E.A., Zhivotovsky B., Lavrik I.N. (2017). Post-translational Modification of Caspases: The Other Side of Apoptosis Regulation. Trends Cell Biol..

[28] Mieyal J.J., Chock P.B. (2012). Posttranslational modification of cysteine in redox signaling and oxidative stress: Focus on s-glutathionylation. Antioxid. Redox Signal.

[29] Matsui R., Ferran B., Oh A., Croteau D., Shao D., Han J., Pimentel D.R., Bachschmid M.M. (2020). Redox Regulation via Glutaredoxin-1 and Protein S-Glutathionylation. Antioxid. Redox Signal.

[30] Gallogly M.M., Starke D.W., Mieyal J.J. (2009). Mechanistic and kinetic details of catalysis of thiol-disulfide exchange by glutaredoxins and potential mechanisms of regulation. Antioxid. Redox Signal.

[31] Bandyopadhyay S., Starke D.W., Mieyal J.J., Gronostajski R.M. (1998). Thioltransferase (glutaredoxin) reactivates the DNA-binding activity of oxidation-inactivated nuclear factor I. J. Biol. Chem..

[32] Pimentel D.R., Adachi T., Ido Y., Heibeck T., Jiang B., Lee Y., Melendez J.A., Cohen R.A., Colucci W.S. (2006). Strain-stimulated hypertrophy in cardiac myocytes is mediated by reactive oxygen species-dependent Ras S-glutathiolation. J. Mol. Cell. Cardiol..

[33] Wang J., Boja E.S., Tan W., Tekle E., Fales H.M., English S., Mieyal J.J., Chock P.B. (2001). Reversible glutathionylation regulates actin polymerization in A431 cells. J. Biol. Chem..

[34] Murata H., Ihara Y., Nakamura H., Yodoi J., Sumikawa K., Kondo T. (2003). Glutaredoxin exerts an antiapoptotic effect by regulating the redox state of Akt. J. Biol. Chem..

[35] Klatt P., Lamas S. (2002). c-Jun regulation by S-glutathionylation. Methods Enzymol..

[36] Frithiof H., Welinder C., Larsson A.M., Rydén L., Aaltonen K. (2015). A novel method for downstream characterization of breast cancer circulating tumor cells following CellSearch isolation. J. Transl. Med..

[37] Brzozowa-Zasada M., Piecuch A., Michalski M., Matysiak N., Kucharzewski M., Łos M.J. (2023). The Clinical Application of Immunohistochemical Expression of Notch4 Protein in Patients with Colon Adenocarcinoma. Int. J. Mol. Sci..

[38] Brzozowa-Zasada M., Ianaro A., Piecuch A., Michalski M., Matysiak N., Stęplewska K. (2023). Immunohistochemical Expression of Glutathione Peroxidase-2 (Gpx-2) and Its Clinical Relevance in Colon Adenocarcinoma Patients. Int. J. Mol. Sci..

[39] Xiong Y., Xiao C., Li Z., Yang X. (2021). Engineering nanomedicine for glutathione depletion-augmented cancer therapy. Chem. Soc. Rev..

[40] Lv H., Zhen C., Liu J., Yang P., Hu L., Shang P. (2019). Unraveling the Potential Role of Glutathione in Multiple Forms of Cell Death in Cancer Therapy. Oxid. Med. Cell Longev..

[41] Chiang F.F., Huang S.C., Yu P.T., Chao T.H., Huang Y.C. (2023). Oxidative Stress Induced by Chemotherapy: Evaluation of Glutathione and Its Related Antioxidant Enzyme Dynamics in Patients with Colorectal Cancer. Nutrients.

[42] Estrela J.M., Ortega A., Obrador E. (2006). Glutathione in cancer biology and therapy. Crit. Rev. Clin. Lab. Sci..

[43] O’Brien M.L., Tew K.D. (1996). Glutathione and related enzymes in multidrug resistance. Eur. J. Cancer.

[44] Piecuch A., Kurek J., Kucharzewski M., Wyrobiec G., Jasiński D., Brzozowa-Zasada M. (2020). Catalase immunoexpression in colorectal lesions. Prz. Gastroenterol..

[45] Piecuch A., Brzozowa-Zasada M., Dziewit B., Segiet O., Kurek J., Kowalczyk-Ziomek G., Wojnicz R., Helewski K. (2016). Immunohistochemical assessment of mitochondrial superoxide dismutase (MnSOD) in colorectal premalignant and malignant lesions. Prz. Gastroenterol..

[46] Nozoe T., Honda M., Inutsuka S., Yasuda M., Korenaga D. (2003). Significance of immunohistochemical expression of manganese superoxide dismutase as a marker of malignant potential in colorectal carcinoma. Oncol. Rep..

[47] Robbins D., Zhao Y. (2014). Manganese superoxide dismutase in cancer prevention. Antioxid. Redox Signal.

[48] Zińczuk J., Maciejczyk M., Zaręba K., Romaniuk W., Markowski A., Kędra B., Zalewska A., Pryczynicz A., Matowicka-Karna J., Guzińska-Ustymowicz K. (2019). Antioxidant Barrier, Redox Status, and Oxidative Damage to Biomolecules in Patients with Colorectal Cancer. Can Malondialdehyde and Catalase Be Markers of Colorectal Cancer Advancement?. Biomolecules.

[49] Gaya-Bover A., Hernández-López R., Alorda-Clara M., Ibarra de la Rosa J.M., Falcó E., Fernández T., Company M.M., Torrens-Mas M., Roca P., Oliver J. (2020). Antioxidant enzymes change in different non-metastatic stages in tumoral and peritumoral tissues of colorectal cancer. Int. J. Biochem. Cell. Biol..

[50] Brzozowa-Zasada M., Piecuch A., Bajdak-Rusinek K., Janelt K., Michalski M., Klymenko O., Matysiak N. (2023). Immunohistochemical Expression of Glutathione Peroxidase 1 (Gpx-1) as an Independent Prognostic Factor in Colon Adenocarcinoma Patients. Pharmaceuticals.

[51] Acevedo-León D., Monzó-Beltrán L., Gómez-Abril S.Á., Estañ-Capell N., Camarasa-Lillo N., Pérez-Ebri M.L., Escandón-Álvarez J., Alonso-Iglesias E., Santaolaria-Ayora M.L., Carbonell-Moncho A. (2021). The Effectiveness of Glutathione Redox Status as a Possible Tumor Marker in Colorectal Cancer. Int. J. Mol. Sci..

[52] Moghadamyeghaneh Z., Hanna M.H., Carmichael J.C., Mills S.D., Pigazzi A., Stamos M.J. (2015). Preoperative leukocytosis in colorectal cancer patients. J. Am. Coll. Surg..

[53] Zeng J., Tang Z.H., Liu S., Guo S.S. (2017). Clinicopathological significance of overexpression of interleukin-6 in colorectal cancer. World J. Gastroenterol..

[54] Jagust P., Alcalá S., Sainz Jr B., Heeschen C., Sancho P. (2020). Glutathione metabolism is essential for self-renewal and chemoresistance of pancreatic cancer stem cells. World J. Stem Cells..

[55] Abdel Hadi N., Reyes-Castellanos G., Carrier A. (2021). Targeting Redox Metabolism in Pancreatic Cancer. Int. J. Mol. Sci..

[56] Nishizawa S., Araki H., Ishikawa Y., Kitazawa S., Hata A., Soga T., Hara T. (2018). Low tumor glutathione level as a sensitivity marker for glutamate-cysteine ligase inhibitors. Oncol. Lett..

[57] Kim A.D., Zhang R., Han X., Kang K.A., Piao M.J., Maeng Y.H., Chang W.Y., Hyun J.W. (2015). Involvement of glutathione and glutathione metabolizing enzymes in human colorectal cancer cell lines and tissues. Mol. Med. Rep..

[58] Oppong D., Schiff W., Shivamadhu M.C., Ahn Y.H. (2023). Chemistry and biology of enzymes in protein glutathionylation. Curr. Opin. Chem. Biol..

[59] Shi T., Dansen T.B. (2020). Reactive Oxygen Species Induced p53 Activation: DNA Damage, Redox Signaling, or Both?. Antioxid. Redox Signal.

[60] Hafsi H., Hainaut P. (2011). Redox control and interplay between p53 isoforms: Roles in the regulation of basal p53 levels, cell fate, and senescence. Antioxid. Redox Signal.

[61] Chen X., Lv Q., Hong Y., Chen X., Cheng B., Wu T. (2017). IL-1β maintains the redox balance by regulating glutaredoxin 1 expression during oral carcinogenesis. J. Oral. Pathol. Med..

[62] Hashemy S.I., Johansson C., Berndt C., Lillig C.H., Holmgren A. (2007). Oxidation and S-nitrosylation of cysteines in human cytosolic and mitochondrial glutaredoxins: Effects on structure and activity. J. Biol. Chem..

[63] Ukuwela A.A., Bush A.I., Wedd A.G., Xiao Z. (2017). Reduction potentials of protein disulfides and catalysis of glutathionylation and deglutathionylation by glutaredoxin enzymes. Biochem. J..

[64] Ogata F.T., Branco V., Vale F.F., Coppo L. (2021). Glutaredoxin: Discovery, redox defense and much more. Redox Biol..

[65] Jung C.H., Thomas J.A. (1996). S-glutathiolated hepatocyte proteins and insulin disulfides as substrates for reduction by glutaredoxin, thioredoxin, protein disulfide isomerase, and glutathione. Arch. Biochem. Biophys..

[66] Ho Y.S., Xiong Y., Ho D.S., Gao J., Chua B.H., Pai H., Mieyal J.J. (2007). Targeted disruption of the glutaredoxin 1 gene does not sensitize adult mice to tissue injury induced by ischemia/reperfusion and hyperoxia. Free Radic. Biol. Med..

[67] Yura Y., Chong B.S.H., Johnson R.D., Watanabe Y., Tsukahara Y., Ferran B., Murdoch C.E., Behring J.B., McComb M.E., Costello C.E. (2019). Endothelial cell-specific redox gene modulation inhibits angiogenesis but promotes B16F0 tumor growth in mice. FASEB J..

[68] Bansal A., Simon M.C. (2018). Glutathione metabolism in cancer progression and treatment resistance. J. Cell Biol..

[69] Peltoniemi M., Kaarteenaho-Wiik R., Säily M., Sormunen R., Pääkkö P., Holmgren A., Soini Y., Kinnula V.L. (2004). Expression of glutaredoxin is highly cell specific in human lung and is decreased by transforming growth factor-beta in vitro and in interstitial lung diseases in vivo. Hum. Pathol..

[70] Reynaert N.L., Wouters E.F., Janssen-Heininger Y.M. (2007). Modulation of glutaredoxin-1 expression in a mouse model of allergic airway disease. Am. J. Respir. Cell Mol. Biol..

[71] Fernandes A.P., Capitanio A., Selenius M., Brodin O., Rundlöf A.K., Björnstedt M. (2009). Expression profiles of thioredoxin family proteins in human lung cancer tissue: Correlation with proliferation and differentiation. Histopathology.

[72] Cha M.K., Kim I.H. (2009). Preferential overexpression of glutaredoxin3 in human colon and lung carcinoma. Cancer Epidemiol..

[73] Nakamura H., Bai J., Nishinaka Y., Ueda S., Sasada T., Ohshio G., Imamura M., Takabayashi A., Yamaoka Y., Yodoi J. (2000). Expression of thioredoxin and glutaredoxin, redox-regulating proteins, in pancreatic cancer. Cancer Detect. Prev..

[74] Abdel-Hamid N.M., Mahmoud T.K., Abass S.A., El-Shishtawy M.M. (2018). Expression of thioredoxin and glutaredoxin in experimental hepatocellular carcinoma-Relevance for prognostic and diagnostic evaluation. Pathophysiology.

[75] Peña C.A., González R., López-Grueso M.J., Antonio Bárcena J. (2015). Redox Regulation Of Metabolic And Signaling Pathways By Thioredoxin and Glutaredoxin in Nitric Oxide Treated Hepatoblastoma Cells. Redox Biol..

[76] González R., López-Grueso M.J., Muntané J., Bárcena J.A., Padilla C.A. (2015). Redox regulation of metabolic and signaling pathways by thioredoxin and glutaredoxin in NOS-3 overexpressing hepatoblastoma cells. Redox Biol..

[77] Brancaccio M., Russo M., Masullo M., Palumbo A., Russo G.L., Castellano I. (2019). Sulfur-containing histidine compounds inhibit γ-glutamyl transpeptidase activity in human cancer cells. J. Biol. Chem..

[78] Fortea-Sanchis C., Martínez-Ramos D., Escrig-Sos J. (2018). The lymph node status as a prognostic factor in colon cancer: Comparative population study of classifications using the logarithm of the ratio between metastatic and nonmetastatic nodes (LODDS) versus the pN-TNM classification and ganglion ratio systems. BMC Cancer.

[79] Remmele W., Stegner H.E. (1987). Vorschlag zur einheitlichen Definition eines Immunreaktiven Score (IRS) für den immunhistochemischen Ostrogenrezeptor-Nachweis (ER-ICA) im Mammakarzinomgewebe [Recommendation for uniform definition of an immunoreactive score (IRS) for immunohistochemical oestrogen receptor detection (ER-ICA) in breast cancer tissue]. Pathologe.

[80] Alnuaimi A.R., Bottner J., Nair V.A., Ali N., Alnakhli R., Dreyer E., Talaat I.M., Busch H., Perner S., Kirfel J. (2023). Immunohistochemical Expression Analysis of Caldesmon Isoforms in Colorectal Carcinoma Reveals Interesting Correlations with Tumor Characteristics. Int. J. Mol. Sci..

